# Clinical Aspects of HyFoSy as Tubal Patency Test in Subfertility Workup

**DOI:** 10.1155/2019/4827376

**Published:** 2019-07-08

**Authors:** Niek Exalto, Mark Hans Emanuel

**Affiliations:** ^1^Division of Obstetrics and Prenatal Medicine, Department of Obstetrics and Gynaecology, Erasmus MC, University Medical Centre, Rotterdam, Netherlands; ^2^Division of Woman and Baby, Department of Gynaecology and Reproductive Medicine, University Medical Centre, Utrecht, Netherlands; ^3^Department of (Uro)gynaecology, University Hospital, Ghent, Belgium

## Abstract

**Background:**

Tubal patency testing is an essential part of female subfertility evaluation. Traditionally, hysterosalpingography (HSG) was the first step to evaluate tubal patency. However, during the past decade Hysterosalpingo-Contrast Sonography (HyCoSy) was introduced in order to avoid radiation exposure and Hysterosalpingo-Foam Sonography (HyFoSy) has been developed as a safe and less painful alternative.

**Objectives and Rationale:**

The aim of this narrative review is to provide an overview of the currently available HyFoSy literature and related clinical aspects.

**Search Methods:**

A literature search was conducted using PubMed and Embase from the introduction of HyFoSy to March 2019. Unfortunately, a meta-analysis was not possible due to a too small number of studies, being mutually incomparable for the various subjects of clinical aspects, even for the reliability as a test for tubal patency.

**Outcomes:**

Nine small studies concluded that the accuracy and effectiveness as a test for tubal patency of 2D- and 3D-HyFoSy are comparable or even better than HSG or HyCoSy. With or without using Doppler techniques, 3D-HyFoSy does not seem to offer benefits above real-time 2D-HyFoSy. Five studies reported on pain and discomfort during HyFoSy, concluding that HyFoSy is a well-tolerated, less painful procedure compared to HSG, without a need for the use of analgesics. There are suggestions about an increased pregnancy rate in the first three cycles after the procedure but in no studies pregnancy outcome after HyFoSy was compared with other or no intervention.

**Wider Implications:**

HyFoSy is a promising and safe alternative for HSG with regard to accuracy and effectiveness. HyFoSy lacks radiation and iodine exposure and is a well-tolerated and less painful procedure than HSG, without the need for analgesics. However more research is needed to make clear statements regarding a therapeutic effect of HyFoSy.

## 1. Introduction

Tubal patency testing is an essential part of female subfertility evaluation [1]. Tubal abnormalities are seen in 30-40% of female subfertility patients [2]. Laparoscopy (LSC) with chromopertubation is considered to be the gold standard diagnostic procedure [3]. LSC is an invasive test, with a risk of intra-abdominal bleeding, visceral damage, and risks related to general anaesthesia. Traditionally, hysterosalpingography (HSG) is a less invasive first-step alternative, which has been used for decades. Considering the disadvantages of HSG such as radiation exposure, discomfort, or even abdominal pain, Hysterosalpingo-Contrast Sonography (HyCoSy) was introduced as an alternative [4, 5] using either contrast media or saline. The accuracy of HyCoSy is comparable to that of HSG and LSC [6, 7] being reference standards for tubal patency testing. An advantage of ultrasound is that, in addition to tubal patency, information is also obtained about pelvic anatomy including uterus and ovaries. Avoiding pregnancy in the treatment cycle is extremely important in HSG procedures.

There are indications that the accuracy of HyCoSy may improve by using 3D imaging techniques [8, 9]. Recently it was claimed in a LSC controlled study [10] that not only tubal patency but also peritubal adhesion could be assessed with HyCoSy by observing a lower “spray score” at the fimbrial end. In a large systematic review with meta-analysis [11] no benefit was found of commercially available contrast media over saline and Doppler sonography was associated with a greater sensitivity and specificity.

However, hyperechogenic contrast media such as Echovist® (Schering AG, Berlin) and SonoVue® (Bracco, Milan) are either no longer available or not licensed for tubal patency testing. Hysterosalpingo-Foam Sonography (HyFoSy) was developed as an alternative for contrast HyCoSy and was introduced in 2010 [12] as a first-line office tubal patency test. Foam is used in the HyFoSy technique to visualize the Fallopian tubes and is created by rigorously mixing 5ml ExEm®-gel (containing hydroxyethyl cellulose and glycerol, IQ Medical Ventures BV, Rotterdam, The Netherlands) with 5 ml purified ExEm®-water. The ExEm®-foam, with a viscosity of 270 cP and containing 94.12 % water, is sufficiently fluid to pass the Fallopian tubes and in the meantime sufficiently stable to show echogenicity for at least five minutes, which is an advantage over saline. In a review on safety aspects and side effects of ExEm-gel [13] it was considered to be appropriate and safe for tubal patency testing. This was confirmed in a recently published retrospective study [14] among 155 women undergoing HyFoSy, reporting no side effects at the follow-up appointment.

This review on tubal patency testing is intended as an update of the literature focusing on clinical aspects of HyFoSy in subfertility workup, like diagnostic accuracy and side effects, 3D and Doppler flow techniques, discomfort and pain, intravasation, pelvic inflammatory disease prevention, and enhancing the chance of pregnancy.

## 2. Methods

A literature search was conducted using PubMed and Embase from the introduction of HyFoSy to 12 March 2019. We searched with terms related to the index test HyFoSy and did not use any filter to maximize the sensitivity of the search. Studies on the use of HyFoSy to confirm occlusion after tubal sterilization were excluded.

Unfortunately, a meta-analysis was not possible due to a too small number of studies, being mutually incomparable for the various subjects of clinical aspects, even for the reliability as a test for tubal patency. We therefore decided to provide a description of published findings and facts as an update of the literature on this subject. The results are summarized in a table containing references per subject, type of the study, study design, and main results (Table 1).

## 3. Diagnostic Accuracy and Side Effects

The first report on efficacy of HyFoSy [15] was a prospective observational cohort study in 73 subfertile women undergoing a HyFoSy. A successful procedure was performed in 67 (92%) of these 73 women. In 57 women (78%) tubal patency was observed and no further examination was needed. In 5 women (7%) tubal occlusion was confirmed by HSG and in another 5 women (7%) there was a discrepancy between HSG and HyFoSy findings. Five patients experienced vasovagal discomfort during or after the procedure that resolved spontaneously in time. No serious adverse effects occurred. In the second observational cohort study [16], comparing the results of 20 HyFoSy procedures with LSC, there was a 100% agreement between HyFoSy and LSC.

In a randomized controlled selective crossover trial (n=40) comparing HyFoSy with saline HyCoSy [17], the proportion of Fallopian tubes that were classified as patent was higher in the HyFoSy group compared to saline HyCoSy (70,0% versus 40,0%, p=0.01). On crossover testing HyFoSy also performed better than saline HyCoSy. No major postprocedural complications were observed.

In a randomized controlled study in 37 infertile women scheduled for LSC [18], the results of HyFoSy and saline HyCoSy were compared with LSC findings. Tubal patency was in the HyFoSy group concordant with LSC in 94,4% of cases (sensitivity 87,5% and specificity 100%) compared to 57,8% in the saline HyCoSy group (sensitivity 50% and specificity 66,6%). In a prospective observational study [19] in 132 women HyFoSy and HyFoSy with High Definition Flow (HDF) Doppler technique was compared to saline HyCoSy and LSC as gold standard. Saline HyCoSy and HyFoSy were in comparison to LSC both significantly less accurate (84.2%; p<0.01, respectively, 92.1%; p<0.01) whereas HyFoSy with HDF Doppler did not significantly differ from LSC (95,8%; p<0.07). Although HyFoSy with and without HDF Doppler technique performed better, the authors conclude that saline HyCoSy may be used as an initial test because of its high negative predictive value on tubal occlusion (99.6% versus 99.5%, respectively; 99% for HyFoSy without HDF Doppler technique).

It is good to realize that it is easier to diagnose tubal patency than tubal occlusion due to a difficult differentiation between true and false occlusion caused by, for example, a mucus plug or a spasm [20]. Actually there is no diagnostic test for tubal occlusion, only for tubal patency.

## 4. 3-Dimensional Ultrasound and Doppler Flow Techniques

HyCoSy with saline (and air) is, in comparison to HyFoSy, more observer-dependent due to the fact that the hyperechoic characteristics are usually lost within a short time and the inability to examine the whole course of the Fallopian tube in one scanning plane [7]. In attempts to overcome these problems 3-dimensional ultrasound (3D-US) and Doppler techniques [21] were introduced. As the hyperechoic characteristics of HyFoSy are much more stable, there is more time for routine 2D ultrasound. It is therefore questionable whether these techniques are of additional value for HyFoSy.

In a prospective observational study [22] in 132 subfertile women, all women underwent HyFoSy with new automated 3D coded contrast imaging (CCI) software (GE Healthcare) with two foam injections followed by 2D real-time HyFoSy. Because the ExEm-foam was too viscous to pass through the 5 French HyFoSy balloon catheter, the investigators prepared a different and unusual off-label ExEm-gel dilution. The concordance rate between the first and second 3D volume reconstruction and the final 2D real-time evaluation was 84.8% and 97.0%, respectively. Vasovagal reactions were observed in two patients and no other adverse effects were noted.

Riganelli et al. [23] described in a pilot study the results of a randomized controlled trial in 50 women who were previously subjected to LSC and randomly assigned to 2D-HyFoSy or 3D-HyFoSy. The 2D-HyFoSy was in 81% of the cases concordant with the LSC, with a sensitivity of 80% and a specificity of 92%. The 3D-HyFoSy was in 88% concordant with LSC, with a sensitivity of 98% and a specificity of 91.4%. Statistical analysis of this difference is lacking in this study. The 3D-HyFoSy procedures were found to be less painful and faster (P<0.001). In the discussion the authors state that 3D-HyFoSy is less operator-dependent and more reproducible and it allows postprocedural reconstruction of images. They conclude that, in low risk patients, if the tubes appear obstructed in 2D-HyFoSy, 3D-HyFoSy seems advisable before submitting patients to LSC.

Ludwin et al. [24] concluded from their retrospective study in 50 women that the interobserver reliability and agreement on the diagnosis of tubal patency using stored videos improved when HyFoSy combined with power Doppler technique was used in comparison to 2D-HyCoSy. The relatively small number of patients in this retrospective study and the lack of real-time 2D information are limitations.

**Figure 1:**
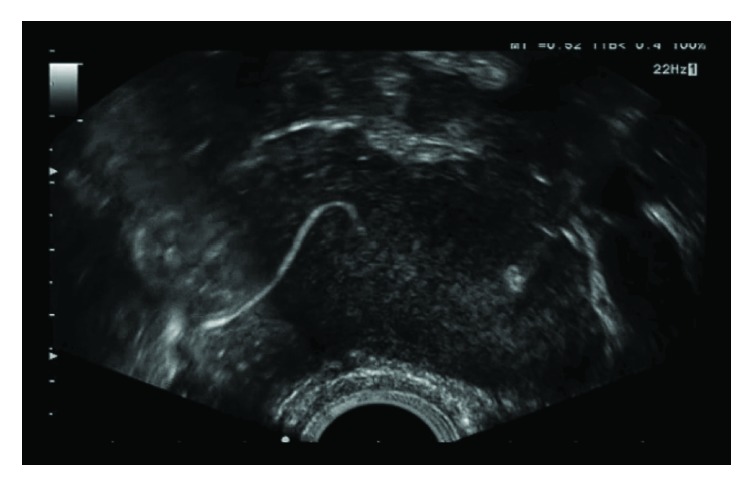
An example of 2D HyFoSy. The clear white line is sufficient evidence for an open Fallopian tube.

With or without using Doppler techniques, 3D-HyFoSy does not seem to offer benefits above real-time 2D-HyFoSy performed by an ultrasonographer who is familiar with pelvic anatomy (Figure 1). The clear white line indicating foam passage through the proximal part of the tube is sufficient evidence for the absence of a distal occlusion as is known from studies using hysteroscopy and air bubbles [25]. Anyway, from the nine clinical studies [15–24], it can be concluded that HyFoSy appears to be accurate and well-tolerated first-line diagnostic procedure and the 3D-HyFoSy technique is helpful for a less experienced operator. Furthermore, 3D scanning offers standardization of pelvic scanning and its use is superb for educational purposes. Recently a novel concept named Fertilityscan© was described [26] using 3D-HyFoSy as a women-friendly and cheap assessment for both anatomy and function of the uterus, ovaries, and tubes.

## 5. Discomfort and Pain

Intrauterine application of contrast media may cause discomfort and pain. This may be due to the dilatation of the cervix, application of a catheter, filling of the cavity under pressure, the composition of the contrast medium, or a combination of factors. In an observational study [27] on 483 patients undergoing saline HyCoSy via a paediatric balloon catheter no pain was observed in 30.0%, mild pain in 49.7%, moderate pain in 13.5%, and severe pain in 6.8% and vasovagal reactions were seen in 4.9%.

In general, a gynaecological examination is for the majority of patients embarrassing and stressful, enhanced by fear or pain [28]. It is important to explain the procedure before starting the examination, to insert the speculum slowly, and, most importantly, to inject the medium very slowly to avoid high intrauterine pressure.

Five studies have been published regarding pain experience during HyFoSy. The first [29] was a randomized controlled trial including 40 women, comparing visual analogue scale (VAS) pain score during tubal patency testing using HyFoSy and serial HSG. For HyFoSy, a small cervical balloonless catheter was placed. For HSG, a hysterophore with one tenaculum on the anterior cervical lip was used. All media were injected in the uterine cavity with the use of an electrical pump with a standardized flow and pressure. This trial showed a lower VAS score in the HyFoSy group compared to the HSG group (median VAS score 1.7 cm; interquartile range (IQR) 4.2 versus median VAS score 3.7 cm; IQR 3.0; P<0.01).

In a cross-sectional study [30] on 216 patients the median VAS score for pain during transvaginal ultrasound (TVU) and subsequent HyFoSy was 1.5 cm (95% CI, 1.2-1.7) and 3.6 cm (95% CI, 3.0-4.0), respectively. To instil the foam, a balloon catheter was placed. One in three women reported the same level of discomfort or pain during TVU as for HyFoSy; 48% of the women reported the HyFoSy to be neutral/unpleasant, but not painful. There was an inverse association between both patient's age and parity and experienced pain [30].

In a randomized controlled trial [31] including 40 women, HyFoSy was performed using two different balloon catheters, uVue HSG® catheter or a paediatric Foley's catheter. It was concluded that a paediatric Foley catheter was easier to insert and the HyFoSy was less painful compared to HyFoSy using uVue HSG® catheter.

One study has been published [32] concerning the use of analgesics during 2D/3D Doppler HyFoSy. In a prospective observational study 300 women were subjected to uterine cavity and tubal patency ultrasound assessment and asked to report VAS pain scores. From October 2012 until March 2013 the procedure was performed without any analgesics in 125 women. From March until the end of the study 175 women received approximately 1 hour before the procedure a tablet containing paracetamol 500mg and codeine phosphate 30mg. During 2D/3D-Doppler-HyFoSy, the median VAS pain score was significantly higher for women not using analgesics (median VAS score 2.0 cm; IQR 1.0-3.0 versus a median VAS score 3.0 cm; IQR 1.3-4.0; p= 0.002).

There are no other studies concerning pain and the placement of the catheter, filling of the uterus, and local or systemic anaesthesia during HyFoSy. We therefore will report on these aspects in relation to gel instillation sonography (GIS), saline infusing sonography (SIS), HSG, and HyCoSy.

### 5.1. The Use of Catheter

It has been suggested [33] that SIS performed with an infant feeding tube without a balloon is associated with very low pain levels in comparison to catheters with a balloon (median pain score 10, on a scale of 0-100). That is consistent with the theory that most sensory receptors are located in the area of the cervical internal ostium. On the other hand, Dessole et al. [34] compared six different catheters, used in 568 sonohysterograms. The diameter of the catheters varied from Charrier 5 to Charrier 8, five had a balloon at the tip with a capacity of 3-5mL, and one was equipped with a movable stopper, which is fixed to the external os of the uterus. The authors did not find significant differences with regard to reliability, the physician's ease of use, the insertion time, the volume of contrast medium, and pain.

### 5.2. Type and Temperature of Contrast

The influence of the contrast medium (Echovist® versus saline) and the temperature (25°C versus 37°C) was investigated in a prospective randomized HyCoSy study [35] including 138 patients. Echovist® induced significantly less pain in comparison to saline at the same temperature (p=0.002 and p=0.001). Between the two groups there was also a significant difference in pain during the introduction of the same contrast at different temperature (p<0.001). The most tolerable one for the patient is body temperature. This applies for both contrast media. These results may indicate that Echovist® is more “patient-friendly” than saline. In another study [36] on pain experience during SIS (n=99) it was observed that postmenopausal women experienced pain more often than premenopausal women (71% versus 32%; p<0.002). Also the character of the pain in relation to saline was different: postmenopausal women more often felt a sharp pain (42%), whereas premenopausal women more often felt gnawing and/or cramping pain (21%). This may be related to the thin atrophic endometrium in postmenopausal women. Furthermore, in a randomized study on 200 HSG procedures [37] it was observed that warming the contrast to body temperature is associated with less pain and fewer vasovagal episodes.

### 5.3. Analgesics

In a randomized double-blinded placebo-controlled trial [38] 127 women received 3 mL 2% lidocaine solution or 0.9% normal saline before undergoing a HSG. As there were no differences with respect to pain scores between both groups, intrauterine lidocaine did not appear to be effective. In a smaller study of 106 women [39], a beneficial effect of intrauterine lidocaine was only seen in parous women undergoing SIS. In a study on 132 patients [40] undergoing GIS with and without lidocaine containing gel prior to a hysteroscopy no differences were seen either. From another randomized double-blind placebo-controlled trial [33] on 120 patients undergoing SIS it could be concluded that topical or local intrauterine application of lidocaine was not effective in reducing pain.

From a systematic review on pain relief in HSG [41] it was concluded that topical analgesics applied before the procedure may be effective, although the available evidence was of low quality. Also, intravenous opioids may be effective though this must be weighed against their side effects. In other systematic reviews and meta-analysis [42, 43] it was concluded that there is no evidence of significant benefit in using any analgesia before HyCoSy or HSG compared to placebo.

Mechanical distension of the uterine walls may cause a release of prostaglandins, resulting in uterine cramps. However in a large randomized double-blind clinical trial [44] (n=816) there was no difference in pain scores between a group receiving an antispasmodic drug (hyoscine-N-butylbromide) during HyCoSy or a placebo. In another study [45] administration of 1000 mg paracetamol and 600 mg ibuprofen one hour prior to office hysteroscopy did not reduce pain scores. Rectal indomethacin, however, reduced the pain significantly during HSG in a randomized placebo-controlled trial [46].

With regard to discomfort and pain it can be concluded from all available literature that HyFoSy is a well-tolerated procedure, less painful than HSG. There is no role for local and general analgesia. Prophylactic analgesia is not necessary and, in case it is nevertheless considered, rectal application of Indomethacin or codeine tablets seems to be effective.

## 6. Intravasation

Venous intravasation is a well-known complication of HSG, occurring in about 6.4% of cases [47]. Rarely cerebral and pulmonary oil embolism after oil-soluble contrast media (OSCM), like Lipiodol®, has been described in case reports and after water-soluble contrast media WSCM complications like fever, infection, and pain have been described. In a study on HyCoSy with SonVue® intravasation occurred in 13.04% of 276 patients [48]. The incidence of intravasation was high in case of thin endometrium and high pressure and low on days 5-7 after ending of the menstrual period. Recently a first case on intravasation during HyFoSy has been published [49]. As hydroxyethyl cellulose and glycerol are safe, even in case of intravenous application [13], no clinical signs or complications occurred.

## 7. Pelvic Inflammatory Disease Prevention

As described in the first paragraph, no major postprocedural complications after HyFoSy were observed in nine observational studies [15–24]. Glycerol, one of the components of ExEm-gel, is known to have antimicrobial and virucidal effects [50]. Fever and peritonitis occurred in only 0.95% of 1.153 patients undergoing sonohysterosalpingography [51]. From HSG studies [52] it is known that only patients with an existing hydrosalpinx are at risk of PID and may benefit from prophylactic antibiotics. Age under 25 years, first sex at an early age, lower socioeconomic status, and exposure to chlamydia trachomatis are risk factors for PID [53].

Routine antibiotic prophylaxis is not beneficial and is not recommended in diagnostic hysteroscopy, because of the very low risk of infection [54–56]. In a Cochrane Review on antibiotics for transcervical intrauterine procedures [57] it was concluded that no trials were eligible for inclusion and it is therefore not possible to draw any conclusion. In a large systematic review on antibiotic prophylaxis for gynaecological procedures prior to and during the utilization of assisted reproductive technologies [58] it is also concluded that routine antibiotic prophylaxis is generally not recommended for these procedures. However, patients at risk of pelvic infections should be screened and treated prior to procedures such as HSG, SIS, HyCoSy, HyFoSy, hysteroscopy, embryo transfer, and chromopertubation.

## 8. Enhancing the Chance of Pregnancy?

Up until now, only observational studies are available concerning the chance of subsequent pregnancy after HyFoSy. A retrospective study [59] reported on a 55% pregnancy rate in 359 women after HyFoSy during a variable follow-up period of 3 to 42 months. In this study the number of pregnancies was the highest in the cycle of the HyFoSy and the first two cycles after the procedure. In a retrospective cohort study [14], among 111 subfertile women, 48 (43.2%) women conceived within 6 months after HyFoSy, of whom 24 women conceived naturally. Emanuel et al. [15] reported a natural conception rate of 19.2% with a median of 3 months after the HyFoSy procedure. In a retrospective observational study [60] regarding 294 subfertile women who underwent HyFoSy, 157 women provided information by phone on their fertility after 12 months. The authors observed a cumulative spontaneous pregnancy rate of 10.2% within 1 month after HyFoSy, 29.9% within 6 months, and 34.4% within 12 months.

More is known about the fertility enhancing effect of tubal flushing at HSG [61]. Tubal flushing with (OSCM) is increasing the odds of pregnancy and live birth in comparison to no intervention or WSCM [62]. It is uncertain whether this is a “tubal flushing” phenomenon, an effect on the intraperitoneal environment, or an implantation enhancing effect on the endometrium. Flushing with an OSCM has been proven to be effective in endometriosis-related infertility [63]. In a recent Cochrane Review [64] the efficacy of tubal flushing with OSCM or WSCM was evaluated. In comparison to no intervention the OSCM group had a higher rate of life birth (OR 3.09, 95% CI 1,39–6.91) compared to the WSCM group (OR 1.13, 95% CI 0.67–1.91). Recently a multicentre RCT on 1119 patients [65] showed significantly more ongoing pregnancies in the first 6 months following HSG with OSCM as compared to HSG with WSCM (39% versus 29%, RR 1.38; 95% CI, 1.17 to 1.64; P<0.001). The increased number of pregnancies in this study was found to be based on pain experienced during the procedure [66]. The use of OSCM in HSG procedures is associated with the occurrence of peritoneal granulomata [67], neonatal hypothyroidism [68], and immunological effects [69].

The clinical impression of enhanced pregnancy rates after HyCoSy with WSCM (Echovist®) could not be confirmed in a prospective randomized study [70] (n=334). In an observational study on 180 patients after saline HyCoSy [71] a possible beneficial effect of HyCoSy was observed directly after the procedure. The pregnancy rate was significantly higher in the first 30 days after HyCoSy (45%) compared to other months of observation after HyCoSy (p<0.0005). One has to keep in mind however that this is comparable to a 42% natural conception rate observed [72] in the first cycle of the normal population. In a retrospective study [73] on 559 patients treated with intrauterine insemination (IUI) it was observed that the cumulative pregnancy rates (mean 2,3 cycles) after LSC, saline HyCoSy, and HSG were 30%, 41%, and 38%, respectively. In a recent post hoc analysis of a prospective multicentre cohort study [74] among 4556 couples with unexplained infertility HSG increased the ongoing pregnancy rate compared to no HSG (adjusted hazard ratio 1.40, 95% CI 1.16-1.70) regardless of WSCM or OSCM was used. Furthermore, in a large study on 1008 infertile patients patency of both Fallopian tubes and the absence of injective resistance turned out to be independent factors associated with the ability to conceive after HyCoSy [75]. This is consistent with the one-half reduction of clinical pregnancies in a large study on the effect of unilateral tubal abnormalities on the results of intrauterine inseminations [76].

In a prospective randomized controlled trial on intrauterine saline infusion as a form of pregnancy enhancing endometrial injury during IVF cycles in 63 patients with recurrent implantation failure [77] a possible negative effect of saline on reproductive outcomes was observed. In this study a clinical pregnancy occurred in 1 out of the 20 women undergoing intrauterine saline infusion on days 3-5 in the stimulation phase as opposed to 9 out of 43 women without an infusion (p<0.05). In a randomized study [78] comparing uterine bathing with OSCM prior to IVF with IVF alone, no evidence was found of any beneficial effect.

ExEm-foam is safe and even passed the mouse-embryo-test [13]. Although in observational studies we could not find any negative effect on fertility after the HyFoSy procedure, we have to conclude that no studies on postprocedure pregnancy rates are available comparing HyFoSy with other or no intervention.

## 9. Conclusion and Discussion

Although most of the studies presented in this review are small with an observational design, it can be concluded that HyFoSy is a promising alternative for HSG with regard to accuracy and effectiveness. HyFoSy lacks radiation and iodine exposure, which is a benefit in comparison to HSG. With or without using Doppler techniques, 3D-HyFoSy does not seem to offer benefits above real-time 2D-HyFoSy. However, 3D scanning offers standardization of pelvic scanning and may be performed by a less experienced operator. HyFoSy is a well-tolerated and less painful procedure than HSG without a need for the use of analgesics. No serious or severe complications have been reported after more than 350.000 procedures. Routine antibiotic prophylaxis is generally not recommended; however patients at risk at pelvic infections should be screened and treated accordingly prior to the HyFoSy procedure. Moreover, there appears to be no detrimental effect of HyFoSy on fertility and there might even be a beneficial effect in the first three menstrual cycles after the procedure on enhancing pregnancies.

## 10. Future Perspectives

As HyFoSy is still a relatively new tubal patency test, only observational data are available. Therefore, robust randomized controlled trials are needed to draw firm conclusion on the degree of accuracy and effectiveness of HyFoSy and the fertility enhancing effect. As ExEm-foam is not yet FDA-approved, large trials and clinical use of HyFoSy in the US are lacking. In the Netherlands a large randomized controlled study, the so-called FOAM trial, comparing the effectiveness and costs of HyFoSy with HSG, is currently ongoing [79]. In this study subfertile patients (N=1163), who are scheduled for tubal patency testing during their fertility workup, undergo both HSG and HyFoSy in a random order. If the results of both tubal tests are discordant, women will be randomly allocated to either a management strategy based on HyFoSy or a management strategy based on HSG, implicating either a LSC or a strategy that assumes tubal patency. The primary outcome of this trial is an ongoing pregnancy leading to live birth within 12 months after randomization. Recruitment for this trial is expected to be completed in the fall of 2018. Therefore, the results will be available around 2020.

With regard to the fertility enhancing effect randomized controlled trials comparing HyFoSy with other or no intervention are urgently needed.

## Figures and Tables

**Table 1:** A summary of the studies used, grouped per reference and subject, mentioning the type of the study, the study design, and the main results.

**Subject/Reference**	**Type of study**	**Study design**	**Main results**
**accuracy**			

[15] Emanuel et al. 2012	Observational	First observational study (n=73)	Successful procedure 92%

[16] Van Schoubroeck et al. 2013	Observational	HyFoSy versus Laparoscopy (n=20)	Agreement 100%

[17] Lim et al. 2015	RCT	HyFoSy versus saline HyCoSy (n=40)	HyFoSy performed better

[18] Piccioni et al. 2017	RCT	LSC controlled trial HyFoSy versus HyCoSy (n=37)	HyFoSy 94,4% versus HyCoSy 57,8%

[19] Ludwin et al. 2017	Observational	LSC controlled trial HyFoSy versus HyFoSy Doppler and saline HyCoSy (n=132)	HyFoSy (92,1%) and HyFoSy Doppler (95,8%) performed better than saline HyCoSy (84,2%)

**3D US and Doppler flow techn.**			

[21] Soliman et al. 2015	Observational	LSC versus saline HyCoSy Power Doppler flow mapping	Power Doppler saline HyCoSy can be incorporated in routine fertility workup

[22] Exacoustos et al. 2017	Observational	2D HyFoSy compared to automated 3D CCI HyFoSy (n=132)	3D CCI HyFoSy is accurate and safe

[23] Riganelli et al. 2018	RCT	LSC controlled 2D HyFoSy versus 3D HyFoSy (n=50)	3D HyFoSy performed better (88% versus 81%)

[24] Ludwin et al. 2017	Observational	Retrospective 2D Doppler HyFoSy versus 2D HyCoSy	2D Doppler HyFoSy performed better

[26] Lavaillant et al. 2019	Observational	Fertiliscan© including 3D HyFoSy	Anatomy of uterus, ovaries and tubes

**Discomfort and pain**			

[27] Savelli et al. 2009	Observational	HyCoSy with paediatric balloon catheter (n=483)	Pain: no (30,0%), mild (49,7%) and severe (6,8%)

[28] Tur-Kaspa 2012	Opinion	Expert opinion and review	Education in gentle technique
[29] Dreyer et al 2014	RCT	VAS score HyFoSy versus HSG (n=40)	Lower VAS score for HyFoSy (1.7 vs 3.7; p<0.01)

[30] Van Schoubroeck et al. 2015	Cross sectional	VAS score TVU and TVU + subsequent HyFoSy (n=216)	Lower VAS score for TVU (1.5 vs 3.6)

[31] Van Schoubroeck et al. 2015	Randomized trial	VAS score HyFoSy with paediatric Foley's catheter versus uVue catheter (n=40)	Foley's catheter easier to insert uVue catheter less painful

[32] Ludwin et al. 2017	Observational	HyFoSy with and without analgesics (n=300)	VAS score higher without analgesics (3.0 vs 2.0; p=0.002)

catheter			

[33] Yung et al 2016	RTC	Without balloon versus with balloon (n=120)	Without balloon less pain

[34] Dessole et al 2001	Observational	Comparison of 6 different catheters (n=568)	No difference observed

temperature			

[35] Fenzl 2012	Observational	Saline and Echovist temp 25^0^ versus 37^0^ (n=138)	Less pain in case of body temperature

[36] Opolskiene et al. 2015	Observational	Saline (SIS) in premenopausal versus postmenopausal women (n=99)	Postmenopausal more pain (71% vs 32%; p<0.002)

[37] Zhu et al. 2012	Observational	HSG room versus body temperature contrast (n=200)	Less pain with warm contrast

analgesics			

[38] Frishman et al. 2004	RCT	lidocaine or saline before HSG (n=127)	No differences in pain score

[39] Guney et al 2007	RCT	Local lidocaine before SIS (n=106)	Only beneficial in parous women

[40] Van den Bosch et al. 2011	Observational	Local lidocaine before Hysteroscopy (n=132)	No difference

[33] Yung et al. 2016	RCT	SIS with and without lidocaine (n=120)	No difference

[41] Hindocha et al. 2015	Systematic review	Pain relief in HSG	Local analgesics may be effective

[42] Ahmad et al. 2007	Systematic review	Any analgesia in HSG	No benefit

[43] Ahmad et al. 2011	Systematic review	Any analgesia in HyCoSy	No benefit

[44] Moro et al 2012	RTC	Antispasmodic drug in HyCoSy (n=816)	No difference in pain scores

[45] Teran-Alonso et al. 2014	Observational	Paracetamol and ibuprofen prior to hysteroscopy	No reduction of pain scores

[46] Karaman et al. 2016	RTC	Rectal indomethacin prior to HSG	Effective in pain reduction

**Intravasation**			

[47] Onwuchekwa and Oriji 2017	Observational	HSG with WSCM (n=299)	Intravasation in 6.4%

[48] Wang et al. 2018	Observational	Intravasation in HyCoSy with SonoVue (n=276)	Intravasation in 13.04%

[49] Ludwin et al. 2018	Case report	First report on intravasation with HyFoSy	

**PID prevention**			

[51] Dessole et al. 2003	Observational	Sonohysterosalpingography (n=1.153)	Fever and peritonitis in 0.95%

[52] Pittaway et al. 1983	Observational	PID after HSG in tubal occlusion 4/35 (11%) without and 0/56 (0%) with antibiotics	Antibiotics only in case of tubal occlusion

[53] Simms et al. 2006	Case control study	Risk factors associated with PID in 140 cases compared to 105 controls	Age <25, early first sex experience, low socio-economic status and chlamydia exposure

[54] Kasius et al. 2011	RCT	AB prophylaxis for hysteroscopy (n=266 AB vs 365 contr)	PID in AB 0.4% versus contr 0%

[55] Gregoriou et al. 2012	RCT	AB prophylaxis for hysteroscopy (n=364 AB vs 188 contr)	PID in AB 0.57% versus contr 0.53%

[56] Nappi et al. 2013	RCT	AB prophylaxis for hysteroscopy (n=523 AB vs 523 contr)	PID in AB 1.0% versus contr 1.15%; p>0.05

[57] Thinkamrop et al. 2013	Cochrane Syst Rev	AB prophylaxis for transcervical intrauterine procedures	No conclusion possible

[58] Pareira et al. 2016	Systematic review	AB prophylaxis for Gynaecologic procedures.	Not as routine, only in high risk cases

**Enhancing chance of pregnancy**			

[59] Van Schoubroeck et al. 2015	Observational	Retrospective study 3-42 months after HyFoSy (n=359)	Pregnancy rate 55%

[14] Tanaka et al. 2018	Observational	Retrospective cohort study 6 months after HyFoSy (n=111)	Pregnancy rate 43%

[15] Emanuel et al. 2012	Observational	Retrospective study 3 months after HyFoSy (n=73)	Pregnancy rate 19.2%

[60] Exacoustos et al. 2015	Observational	Retrospective study 1, 6 and 12 months after HyFoSy (n=157)	Pregnancy rates 10.2%, 29.6% and 34.4%

[61] Watson et al. 1994	Meta analysis	4 RTCs and 6 others on HSG with OSCM versus WSCM	Therapeutic effect of OSCM

[62] Johnson et al. 2005	Cochrane Syst Rev	Systematic Review OSCM and OCSM versus WSCM	Therapeutic effect of OSCM

[63] Johnson 2014	Narrative review	OSCM treatment of infertility	Therapeutic effect in endometriosis

[64] Mohiyiddeen et al. 2015	Cochrane Syst Rev	The effect of tubal flushing on life birth and pregnancy rates	Higher life birth rate for OSCM versus WSCM (OR 3.09 versus 1.38)

[65] Dreyer et al. 2017	Multicentre RCT	Pregnancy 6 months after OSCM versus WSCM (n=1119)	Higher pregnancy rate after OSCM (39% vs 29%)

[70] Lindborg et al. 2009	RCT	HyCoSy with WSCM (n=334)	No enhanced pregnancy rate

[71] Giugliano et al 2012	Observational	Saline HyCoSy (n=180)	45% pregnancy rate in the first 30 days

[73] Ahinko-Hakamaa et al. 2007	Observational	Pregnancy rates after (mean) 2.3 cycles with IUI.	Pregnancy rates after LSC 30%, HyCoSy 41% and HSG 38%.

[74] Dreyer et al. 2019	Post-hoc analysis	Prospective multicentre cohort study (n= 4556)	HSG increased pregnancy rate compared to no HSG regardless of WSCM or OSCM

[75] Chunyan et al. 2018	Observational	Pregnancy within 180 days after HyCoSy (n=1008)	Higher pregnancy rates if both tubes are open

[77] Salehpour et al. 2016	RCT	Saline infusion prior to IVF (n=20) versus controls (n=39)	Pregnancy 1/20 versus 9/39 (p=0.01)

[78] Reilly et al. 2019	RCT	OSCM endometrial bathing prior to IVF (n=33) vs controls (n=37) in women with endometriosis	Pregnancy within 6 months 11/33 (33%) versus 12/37 (32%)
